# Root enhancement improves rhizosphere nutrient availability and promotes growth in flue-cured tobacco

**DOI:** 10.3389/fpls.2025.1728181

**Published:** 2026-01-15

**Authors:** Linyi Yu, Mingfa Zhang, Sheng Zhang, Minggang Chen, Mouzhi Yuan, Jialing Huang, Wenbo Chen, Yiyang Zhang

**Affiliations:** 1 College of Agronomy, Hunan Agricultural University, Changsha, China; 2 Xiangxi Branch of Hunan Provincial Tobacco Corporation, Xiangxi, China; 3 Zhangjiajie Branch of Hunan Tobacco Corporation, Zhangjiajie, China

**Keywords:** flue-cured tobacco, mediation effect, microbialcommunity, rhizosphere environment, root architecture

## Abstract

**Introduction:**

Enhancing root development can profoundly reshape rhizosphere symbioses that influence nutrient uptake and plant growth. However, the mechanisms linking root optimization, rhizosphere microbial assembly, and nutrient dynamics in flue-cured tobacco remain insufficiently understood.

**Methods:**

A field experiment was conducted using flue-cured tobacco (Nicotiana tabacum L., cv. Yunyan 87) to compare an enhanced-root treatment (nutrient-bag seedling system under alternating moisture) with conventional floating seedling cultivation. Root traits, rhizosphere nutrient availability, soil enzyme activities, microbial community composition, plant nutrient accumulation, and mediation relationships among root traits, rhizosphere environment, and plant growth were evaluated.

**Results:**

The enhanced-root treatment significantly increased root length and root volume (up to 65.6% and 51.5%, respectively). Rhizosphere function was improved, as indicated by higher available phosphorus (+51.7%) and urease activity (+29.6%). Microbial community composition shifted toward beneficial taxa, including enrichment of Rhizobiaceae and Actinobacteria. These changes were associated with greater nutrient acquisition, increasing total nitrogen, total phosphorus, and total potassium accumulation by 13–14%. Mediation analysis further demonstrated that the rhizosphere environment fully mediated the positive effects of root optimization on plant growth, supporting a causal chain of “root system → rhizosphere symbiosis → plant performance.”

**Discussion:**

Structural and functional enhancement of roots strengthens plant–microbe symbiosis and promotes nutrient cycling, thereby improving tobacco growth and nutrient accumulation. These findings provide a mechanistic framework for root-based strategies to enhance tobacco productivity while supporting soil ecological function.

## 1 Introduction

The root system is the core organ of the belowground plant body and the primary interface mediating plant–soil interactions. Beyond their basic roles in water and nutrient uptake, roots regulate the chemical and biological properties of the surrounding soil and thereby exert a strong influence on plant growth and soil ecological functions (Hochholdinger, 2016). By modulating root architecture and activity, it is therefore possible to improve both crop productivity and the sustainability of agroecosystems. Flue-cured tobacco (Nicotiana tabacum L.) is particularly suitable as a model for such investigations, because its yield and leaf quality are highly sensitive to variation in root performance and rhizosphere processes throughout the field growth period (Hu et al., 2021; Ren et al., 2024).

In commercial tobacco production, the seedling-raising stage is a critical window that shapes subsequent field performance. Seedling quality determines transplanting success, early root establishment, and the long-term growth potential of tobacco plants after they are moved to the field (Zhao et al., 2017; Li et al., 2018; Wang et al., 2019). Over the past decades, several seedling-raising systems have been developed, including conventional floating seedling cultivation, various modified shallow-water systems, substrate-based plug trays, and more recently nutrient-bag or pot seedling techniques (Dong et al., 2024; Tao et al., 2024). These approaches have been shown to influence root biomass, root vigor, shoot morphology, and ultimately tobacco yield and quality. Similar observations in other crops indicate that seedling-raising methods can induce persistent differences in root architecture that carry over into the field phase, with important consequences for nutrient acquisition and stress resistance (Guo, 2023; Voothuluru et al., 2024). However, most studies to date have focused on aboveground traits or simple root indices at the seedling stage, and only a few have examined how different seedling-raising methods regulate the rhizosphere environment after transplanting. In particular, there is still a lack of systematic research on how seedling-induced changes in root architecture affect rhizosphere nutrient availability, soil enzyme activities, and microbial communities in flue-cured tobacco.

Root architecture is a key determinant of the formation and functioning of the rhizosphere. Root traits such as total length, surface area, branching density, and root tip abundance influence the quantity and spatial distribution of root exudates, including organic acids, sugars, amino acids, phenolics, and enzymes (Khan et al., 2021; Santoyo, 2022). These exudates can modify soil pH, mobilize poorly soluble nutrients, and regulate the activity and composition of soil microorganisms, collectively creating a dynamic rhizosphere microenvironment (Hu et al., 2018; de la Fuente Cantó et al., 2020; Lin et al., 2022). Rhizosphere microorganisms, in turn, participate in key nutrient cycling processes such as nitrogen mineralization, phosphorus solubilization, and potassium activation, and can produce phytohormones that enhance plant growth and stress tolerance (Wang et al., 2022a; Vincze et al., 2024). Thus, changes in root architecture can simultaneously reshape soil nutrient status, soil enzyme activities, and microbial community structure, which together feedback to influence plant nutrient uptake, biomass accumulation, and yield. This forms a mechanistic chain of “root architecture → rhizosphere environment → plant performance” that provides a useful conceptual framework for evaluating the effects of seedling-raising methods.

To better exploit this framework in tobacco production, new seedling-raising techniques that specifically target root development have been proposed. In our previous nursery-stage work, we developed a nutrient-bag seedling system under an alternating moisture regime. This technique employs degradable non-woven fabric bags filled with a composite substrate to provide a more favorable physical and nutritional environment for root growth than conventional floating seedling cultivation, thereby enhancing root biomass, root activity, and leaf development at the seedling stage. However, it remains unclear how such root-enhancement strategies modulate the rhizosphere environment after transplanting and whether these changes can be quantitatively linked to improvements in tobacco growth.

In this study, we used flue-cured tobacco cv. ‘Yunyan 87’ to compare a conventional floating seedling system with the nutrient-bag seedling cultivation technique that enhances root development. Specifically, we aimed to: (1) characterize the effects of root-enhancement seedling cultivation on root architecture and physiological activity during the field growth period; (2) evaluate the associated changes in rhizosphere soil properties, including pH, nutrient availability, soil organic matter, enzyme activities, and microbial community composition; and (3) clarify how these root- and rhizosphere-level changes contribute to tobacco nutrient uptake, biomass accumulation, and agronomic performance using correlation and mediation analyses. By integrating root traits, rhizosphere indicators, and plant growth responses, we sought to elucidate the regulatory mechanism of the “root architecture–rhizosphere environment–plant growth” system under different seedling-raising methods and provide a theoretical basis for optimizing seedling management in flue-cured tobacco.

## 2 Materials and methods

### 2.1 Experimental site and design

The field experiment was conducted in 2024 in Denggao Village, Dao’er Township, Huayuan County, Xiangxi Tujia and Miao Autonomous Prefecture, Hunan Province, China (28.53°N, 109.45°E). The site is located at an altitude of 530 m and has a humid subtropical monsoon climate, with a mean annual temperature of 15.0°C, annual precipitation of 1365 mm, annual sunshine duration of 1219 h, and a frost-free period of 279 days. The soil is classified as a Haplic Acrisol according to the FAO/WRB system. Before transplanting, composite 0–20 cm soil samples were collected to determine the basic physicochemical properties of the experimental soil (Coller et al., 2022). The soil had a pH of 5.74, organic matter content of 21.46 g/kg, total nitrogen of 1.51 g/kg, hydrolyzable nitrogen of 109.00 mg/kg, total phosphorus of 0.628 g/kg, total potassium of 1.671 g/kg, available phosphorus of 10.94 mg/kg, and available potassium of 154.75 mg/kg (**Table 1**). Two treatments were established: a conventional root development level (CK, floating seedling cultivation) and an enhanced root development level (Nb, nutrient-bag humid seedling cultivation). For clarity, “root enhancement” in this study refers specifically to the nutrient-bag seedling cultivation (Nb) technique, rather than to root morphology itself. In the CK treatment, seedlings were grown in traditional foam floating trays filled with a commercial seedling substrate. In the Nb treatment, a composite substrate consisting of earthworm cast, coconut coir, perlite, and vermiculite mixed at a volumetric ratio of 2:2:3:3 was used to fill degradable non-woven fabric nutrient bags for seedling cultivation. All seedlings were transplanted to the field on 18 April 2024, using a planting density of 120 × 55 cm (row spacing × plant spacing). Fertilization followed a standardized nutrient-supply regime. Basal fertilizer was applied before ridge formation and consisted of 859 kg/ha of tobacco-specific basal fertilizer (N–P_2_O_5_–K_2_O; 8–15–7), one-half of the tobacco-specific topdressing fertilizer (N–P_2_O_5_–K_2_O; 10–5–29), and one-half of the potassium sulfate (N–P_2_O_5_–K_2_O; 0–0–52). A seedling-establishment fertilizer (75 kg/ha; N–P_2_O_5_–K_2_O; 20–9–0) was applied 7 days after transplanting. The remaining one-half of the tobacco-specific topdressing fertilizer and potassium sulfate were applied as topdressing in two split applications within 25 days after transplanting. In total, nutrient inputs corresponded to an N:P _2_O_5_:K _2_O ratio of 1:1.33:3.10. The flue-cured tobacco cultivar used was Yunyan 87, provided by the Huayuan County Branch of the Xiangxi Prefecture Tobacco Company, Hunan Province. Water and fertilizer management during the seedling stage followed standard practices. After transplanting, field management practices, including fertilization, irrigation, weeding, and pest and disease control, were performed according to local tobacco cultivation standards and were kept consistent across treatments.

**Table 1:** Basic physicochemical properties of the experimental soil before transplanting.

pH	Total nitrogen (g/kg)	Hydrolyzable nitrogen (mg/kg)	Total phosphorus (g/kg)	Total potassium (g/kg)	Available phosphorus (mg/kg)	Available potassium (mg/kg)	organic matter (g/kg)
5.74	1.51	109.00	0.628	1.671	10.94	154.75	21.46

### 2.2 Sample collection and processing

Topsoil (0–3 cm) surrounding the tobacco plants was first removed. Then, using a spade, soil was excavated to a depth exposing the rhizosphere approximately 25 cm from the tobacco stem. This procedure was repeated three times for each sampling site (Zhou et al., 2023a). The collected soil samples were immediately placed in polyethylene boxes containing ice packs for temporary storage. Samples were then passed through a 2 mm sieve and homogenized. After homogenization, each soil sample was divided into two subsamples. One subsample was air-dried for physicochemical analyses. The other subsample was kept fresh and separated for biological analyses: soil enzyme assays were performed on material stored at 4°C and analyzed within 48 h, whereas DNA extraction and microbial community analysis used material stored at −80°C. Destructive plant sampling was performed at 30, 60, and 90 days after transplanting (DAT). For each treatment, three representative tobacco plants were selected, and roots, stems, and leaves were oven-dried for biomass determination.

### 2.3 Measurement indicators and methods

#### 2.3.1 Plant agronomic traits and biomass

Plant height, stem girth, number of functional leaves, maximum leaf length, and maximum leaf width were measured following the flue-cured tobacco industry standard YC/T 142-2010, and using approaches consistent with peer-reviewed studies on tobacco agronomic traits (Wang et al., 2024). Plants were separated into roots, stems, and leaves. Fresh samples were first heated at 105°C for 30 min to deactivate enzymes and then oven-dried at 75°C to a constant weight (Man et al., 2016).

#### 2.3.2 Determination of plant nutrient content

Dried plant samples were ground and passed through a 100-mesh sieve. Total nitrogen (N) was determined by the Kjeldahl method (Khan et al., 2025), total phosphorus (P) by the molybdenum–antimony colorimetric method (Wieczorek et al., 2022), and total potassium (K) by flame photometry (Zhai et al., 2013). Nutrient accumulation in each organ and in the whole plant, as well as nutrient distribution ratios among organs, were calculated based on dry weight and nutrient content.

#### 2.3.3 Determination of root morphology and physiological function

Fresh roots were gently washed with deionized water to remove adhering soil. Total root length, root surface area, root volume, number of root tips, and number of branches were measured using a WinRHIZO root scanning analysis system (Chen et al., 2020). Root activity was determined by the TTC (2,3,5-triphenyltetrazolium chloride) reduction method (Man et al., 2016).

For xylem sap collection, the stem was excised approximately 6 cm above the root–shoot junction, rinsed with deionized water, and blotted dry. A sterile plastic container partially filled with dry absorbent cotton was covered with elastic parafilm, and the cut stem end was inserted such that it contacted the cotton surface. Sap was collected for 12 hours (from 19:00 to 07:00 the following morning), following commonly adopted protocols for passive xylem exudation under root pressure (Netting et al., 2012; Alexou and Peuke, 2013).

#### 2.3.4 Determination of soil physicochemical properties and enzyme activities

Soil pH was determined by the potentiometric method. Soil organic matter was determined by the potassium dichromate external heating method. Total nitrogen was measured by the Kjeldahl method; total phosphorus by NaOH fusion followed by the molybdenum–antimony colorimetric method; total potassium by NaOH fusion followed by flame photometry; hydrolyzable nitrogen by the alkaline hydrolysis diffusion method; available phosphorus by the Olsen method; and available potassium by 1 mol/L ammonium acetate (NH_4_OAc) extraction followed by flame photometry (Zhu et al., 2019). Soil enzyme activities were determined as follows: sucrase (invertase, INV) activity by the 3,5-dinitrosalicylic acid colorimetric method; urease (URE) activity by the indophenol blue colorimetric method; and catalase (CAT) activity by the permanganate titration method (Zhang et al., 2020).

#### 2.3.5 Soil microbial community analysis

Total genomic DNA was extracted from 0.5 g of fresh soil using a commercial soil DNA extraction kit following the manufacturer’s instructions. The V4 region of the bacterial 16S rRNA gene was amplified using primers 515F (GTGCCAGCMGCCGCGGTAA) and 806R (GGACTACHVGGGTWTCTAAT). PCR amplification was carried out using Phusion High-Fidelity DNA Polymerase under standard cycling conditions. Amplicons were purified, quantified, pooled in equal molarity and used for library construction. After Qubit and qPCR quality control, libraries were sequenced on an Illumina NovaSeq 6000 platform (PE250).

Raw reads were demultiplexed, merged using FLASH, and quality-filtered with fastp to obtain high-quality tags. Chimeras were removed using the SILVA database as reference. Clean reads were processed using QIIME2, and ASVs were generated with the DADA2 algorithm. Taxonomic classification was performed using SILVA 138.1. Alpha-diversity indices (Chao1, Shannon) and beta-diversity (Bray–Curtis distance) were calculated after rarefaction. Principal coordinate analysis (PCoA) was used to visualize microbial community differences, and differential taxa were identified based on linear discriminant analysis (LDA) using the LEfSe framework, and taxa exceeding the LDA threshold were visualized using non-phylogenetic LDA barplots.

### 2.4 Data processing and statistical analysis

Data were organized using Microsoft Excel 2016. Data analyses were performed using Python, with the significance level set at p < 0.05. Mediation analysis was performed using the Bootstrap method with 5,000 resamples and a random seed of 42. Linear models were used to estimate path coefficients (a, b, c, and c′). Significance was determined using the percentile method with a 95% confidence interval (CI). An indirect effect was considered significant if the CI did not include zero. Complete or partial mediation was distinguished based on whether the CI of the direct effect (c′) included zero.

## 3 Results

### 3.1 Effects of root enhancement on root architecture and physiological activity during the field growth stage

**Figure 1:**
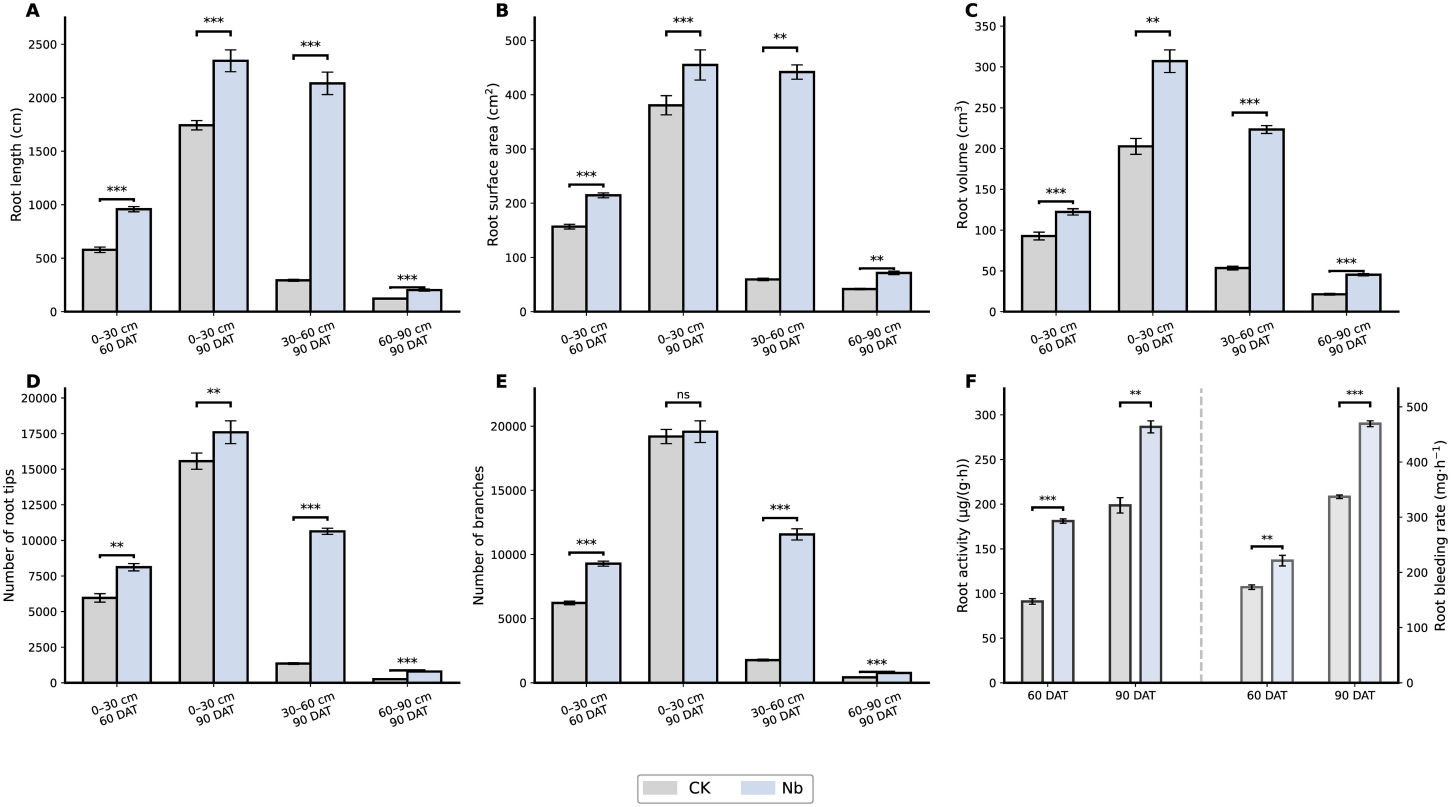
Effects of root enhancement on root structure and function. Changes in **(A)** root length, **(B)** root surface area, **(C)** root volume, **(D)** number of root tips, **(E)** number of branches, and **(F)** root activity and bleeding rate under different treatments. CK represents the control treatment, and Nb represents the treatment group. Error bars indicate standard error (SE). Statistical significance is denoted as **P < 0.01, ***P < 0.001, and ns indicates no significant difference.

The enhanced-root treatment (Nb) markedly promoted both the structural development and physiological activity of the tobacco root system during the field growth period (**Figure 1**). At 60 days after transplanting (DAT), total root length reached 958.32 cm, representing a 65.6% increase compared with the conventional floating seedling control (CK) (P < 0.001). Root surface area and volume increased by 36.6% and 31.9%, respectively, and the numbers of root tips and branches increased by 36.2% and 49.3% (P < 0.01). This structural advantage persisted to 90 DAT, at which time the Nb treatment still exhibited 34.5% higher root length and 51.5% higher root volume relative to CK (P < 0.01). Root physiological activity was similarly enhanced, showing increases of 98.8% at 60 DAT and 44.3% at 90 DAT compared with the control (P < 0.01). Overall, the Nb treatment consistently strengthened both root structural traits and physiological activity throughout the growth period, establishing a strong foundation for subsequent improvements in rhizosphere nutrient availability and plant nutrient acquisition.

### 3.2 Effects of root enhancement on soil physicochemical properties

**Figure 2:**
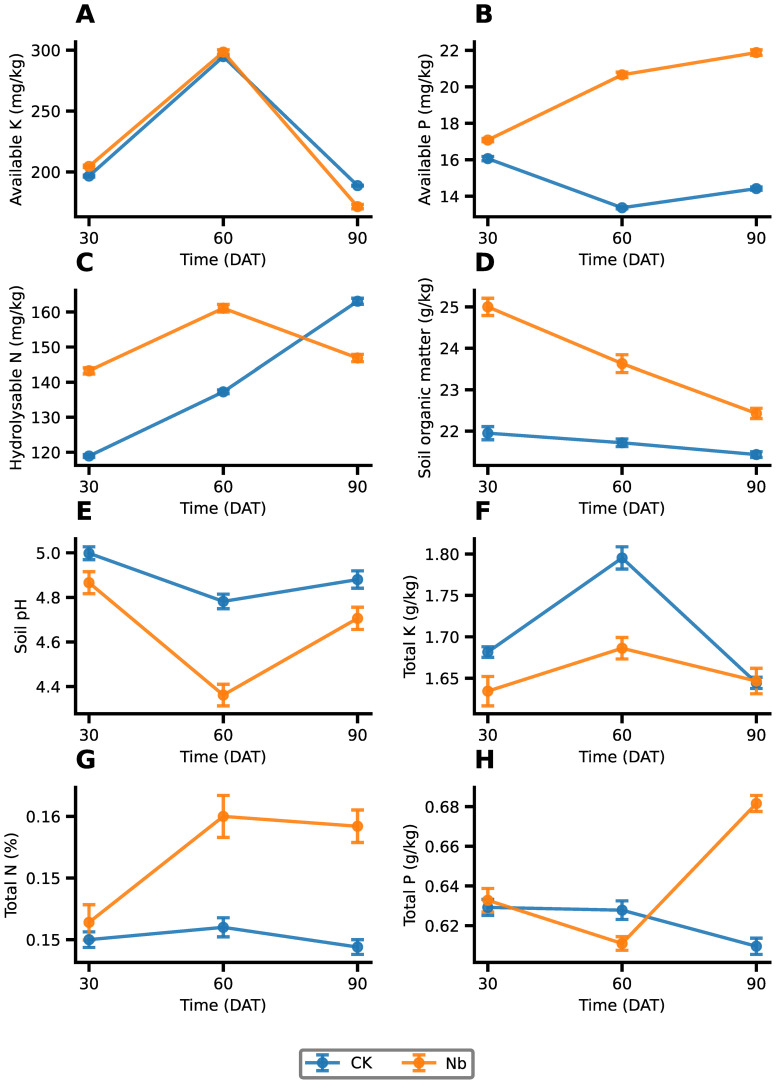
Effects of root enhancement on soil physicochemical properties. Changes in **(A)** hydrolyzable N, **(B)** available K, **(C)** available P, **(D)** soil organic matter content, **(E)** soil pH, **(F)** total K, **(G)** total N, and **(H)** total P under different treatments. CK represents the control treatment, and Nb represents the treatment group. Error bars indicate standard error (SE).

Root enhancement markedly modified the physicochemical conditions of the rhizosphere soil during field growth (**Figure 2**). Available phosphorus increased substantially under the Nb treatment, rising by 54.5% at 60 DAT and 51.7% at 90 DAT (P < 0.001). Hydrolyzable nitrogen also increased by 20.4% and 17.3% at 30 and 60 DAT, respectively (P < 0.01), but fell below the control by 9.9% at 90 DAT, reflecting a shift from nutrient release to rapid plant uptake in later stages. The response of available potassium differed across the growth period. A slight increase was observed at 30 DAT (+4.0%), followed by no significant difference at 60 DAT, and a reduction below CK at 90 DAT (–9.1%). This pattern aligns with intensified potassium uptake by the more developed root system at later stages. Soil pH showed a time-dependent decline under Nb, becoming significantly lower than CK at 60 DAT (P < 0.001), indicating enhanced rhizosphere acidification favorable for nutrient mobilization. Soil organic matter exhibited an early but modest enrichment in absolute terms. At 30 DAT, SOM under Nb was only 3.05 g/kg higher than CK, although this difference appeared as a 13.9% increase due to the moderate baseline level. The Nb–CK gap subsequently declined to 1.91 g/kg at 60 DAT and 0.99 g/kg at 90 DAT. Because sampling targeted the immediate rhizosphere rather than bulk soil, these short-term fluctuations likely reflect transient inputs of root exudates, sloughed tissues, and microbial necromass—components that are readily captured by dichromate oxidation—rather than long-term changes in background SOM. Overall, the Nb treatment created a more biochemically active rhizosphere by enhancing early nutrient availability, promoting acidification, and temporarily increasing labile organic matter inputs.

### 3.3 Effects of root enhancement on plant nutrient uptake and allocation

**Figure 3:**
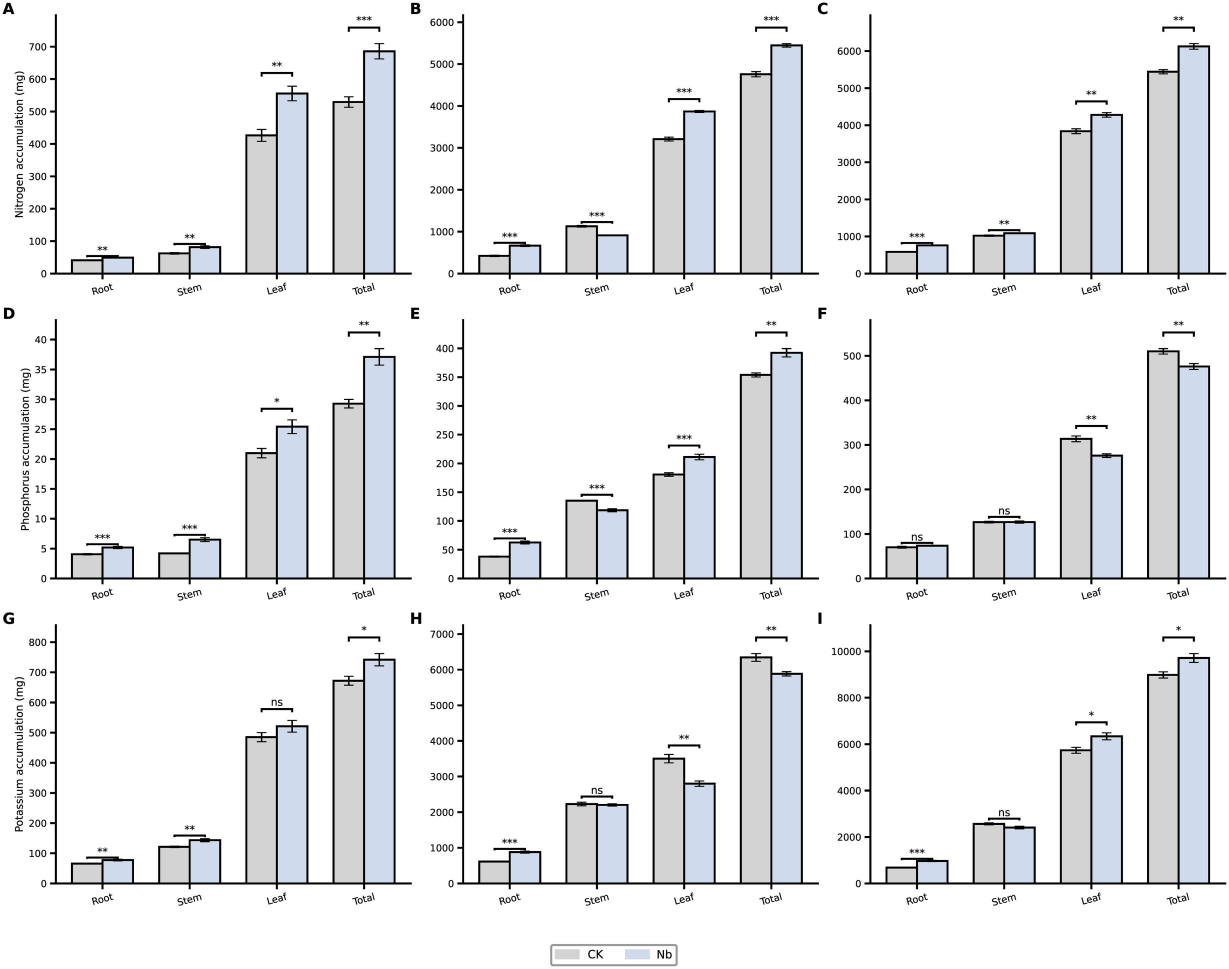
Effects of root enhancement on nutrient uptake and allocation. Accumulation of **(A–C)** nitrogen (N), **(D–F)** phosphorus **(P)**, and **(G–I)** potassium (K) in roots, stems, leaves, and the whole plant under different treatments at 30, 60, and 90 days after transplanting. CK represents the control treatment, and Nb represents the treatment group. Error bars indicate standard error (SE). Statistical significance is denoted as *P < 0.05, **P < 0.01, ***P < 0.001, and ns indicates no significant difference.

**Figure 4:**
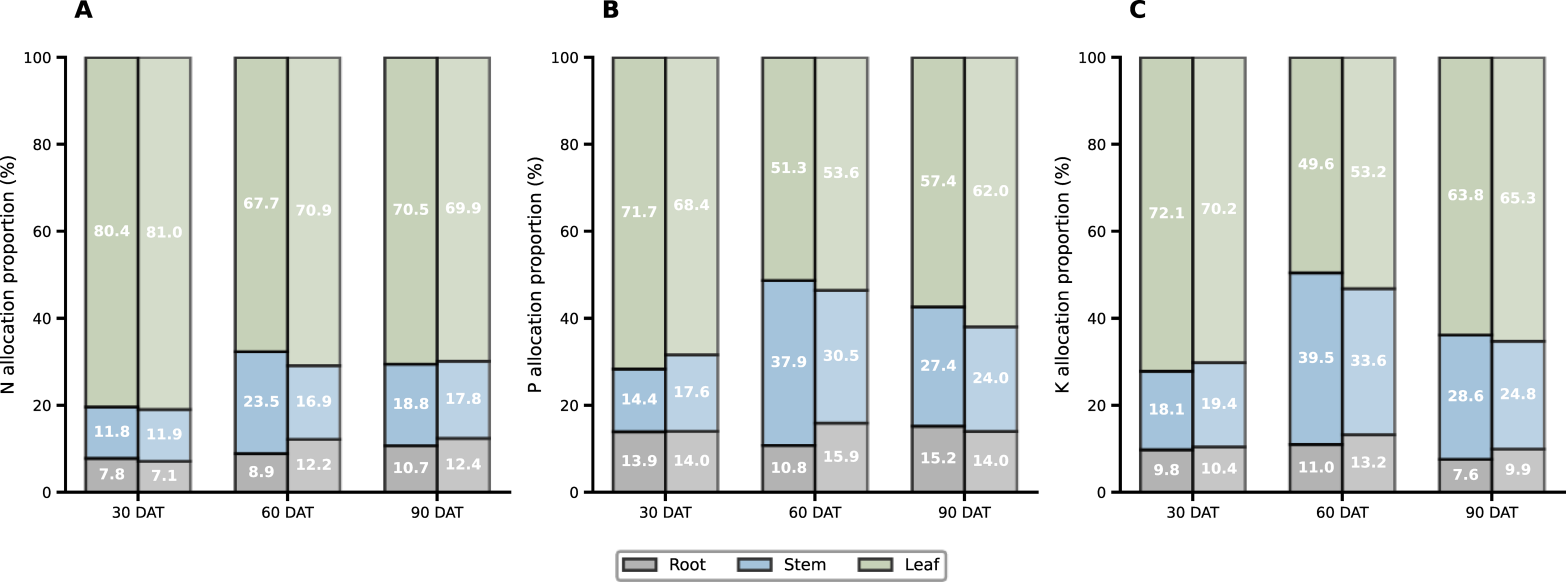
Effects of root enhancement on nutrient allocation. Proportional distribution of **(A)** nitrogen (N), **(B)** phosphorus (P), and **(C)** potassium (K) among roots, stems, and leaves under different treatments at 30, 60, and 90 days after transplanting. For each time point, the left bar represents the CK treatment, and the right bar represents the Nb treatment.

Plant nutrient acquisition increased consistently under the Nb treatment throughout the field growth period (**Figure 3**; **Figure 4**). At 30, 60, and 90 days after transplanting (DAT), total nitrogen accumulation increased by 30%, 15%, and 13%, respectively. Phosphorus accumulation showed a similar trend, rising by 27%, 12%, and 14%, while potassium accumulation increased by 10%, 19%, and 8%, respectively. Root enhancement also altered nutrient allocation patterns within the plant. At 60 DAT, the proportion of nitrogen distributed to the roots increased from 8.87% (CK) to 12.18% (Nb), whereas the proportion allocated to stems decreased from 23.47% to 16.93%. Both phosphorus and potassium exhibited comparable shifts in their allocation ratios, indicating a coordinated adjustment of nutrient distribution in response to improved root development. Overall, the Nb treatment promoted greater nutrient accumulation and supported a more favorable internal allocation pattern, contributing to enhanced physiological performance during the mid- to late growth stages.

### 3.4 Effects of root enhancement on soil enzyme activities and microbial community

**Figure 5:**
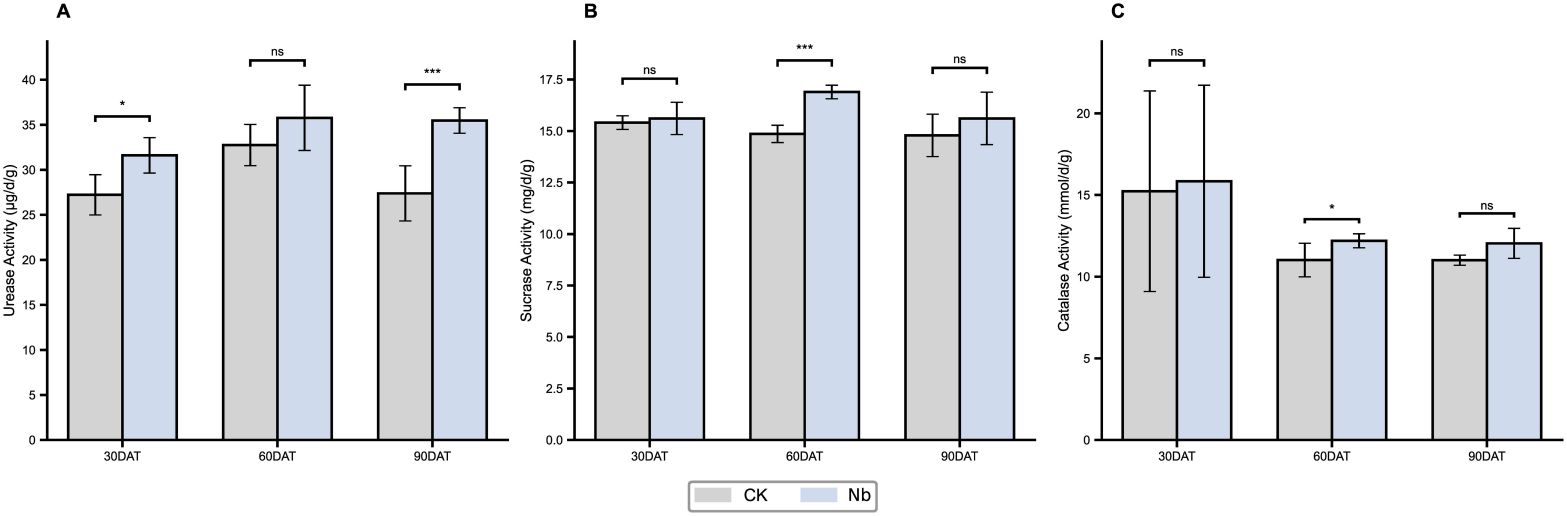
Effects of root enhancement on soil enzyme activities. Changes in **(A)** urease activity, **(B)** sucrase activity, and **(C)** catalase activity in soils under different treatments at 30, 60, and 90 days after transplanting. CK represents the control treatment, and Nb represents the treatment group. Error bars indicate standard error (SE). Statistical significance is denoted as *P < 0.05, ***P < 0.001, and ns indicates no significant difference.

**Figure 6:**
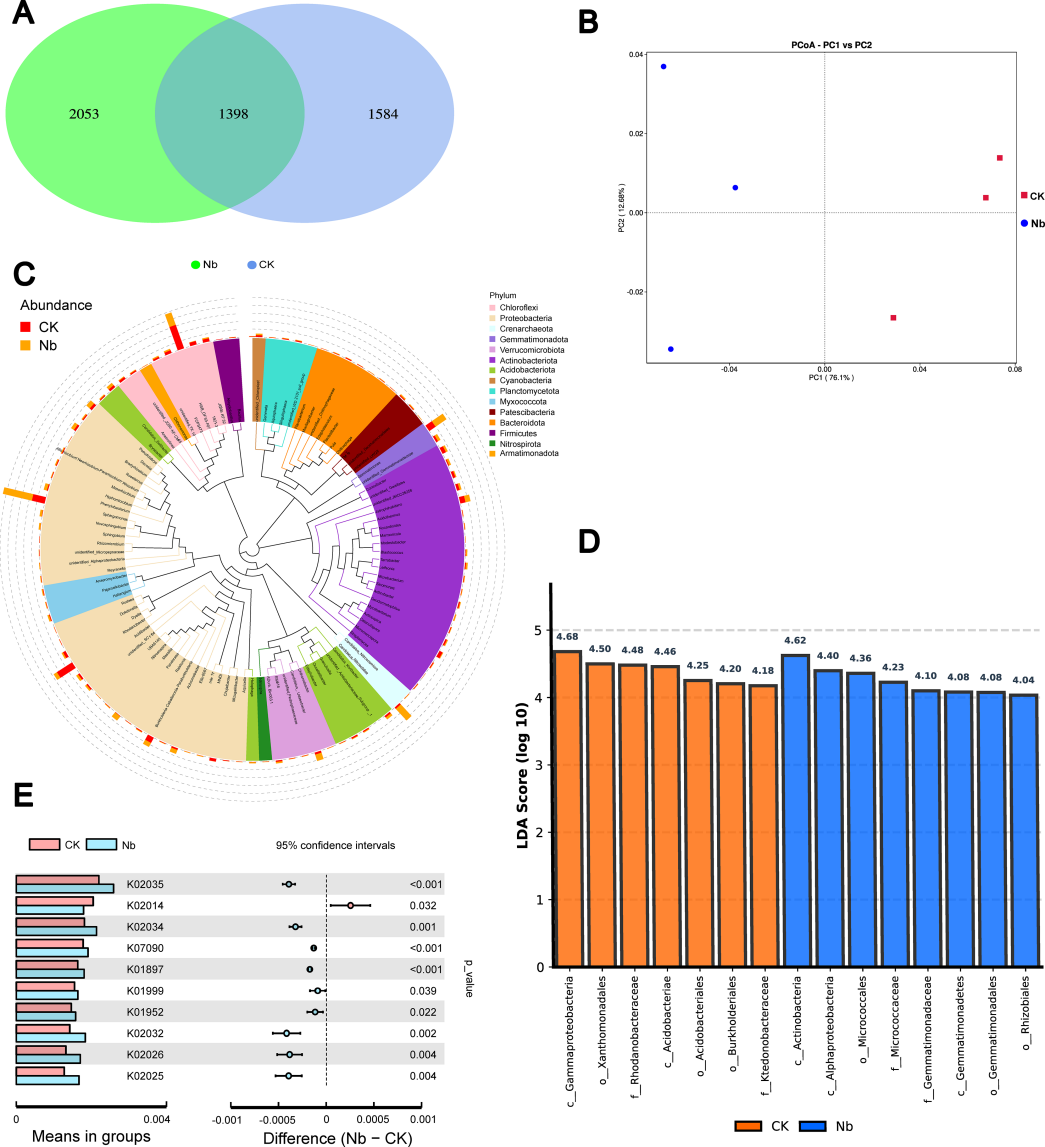
Effects of root enhancement on the structure and functional potential of the rhizosphere bacterial community. **(A)** Venn diagram showing shared and unique bacterial ASVs between treatments. **(B)** Principal coordinates analysis (PCoA) of bacterial community composition based on Bray–Curtis distance. **(C)** Phylogenetic tree showing the overall taxonomic structure of dominant bacterial phyla. **(D)** Differential bacterial taxa between CK and Nb identified by LEfSe (P < 0.05, LDA score > 4.0). **(E)** Predicted functional pathways showing significant differences between treatments.

Root enhancement exerted selective but substantial influences on rhizosphere biochemical activity (**Figure 5**). Urease activity increased by 16.1% at 30 DAT (P < 0.05) and 29.6% at 90 DAT (P < 0.001), indicating an accelerated soil nitrogen transformation process. Sucrase activity showed its greatest response at 60 DAT, rising by 13.7% (P < 0.001), whereas catalase activity exhibited a moderate but significant increase of 10.7% at 60 DAT (P < 0.05). These trends suggest that the Nb treatment enhanced both carbon- and nitrogen-related enzymatic processes, contributing to a more active rhizosphere environment. Changes in microbial community composition further reflected the restructuring of the rhizosphere (**Figure 6**). The Nb treatment increased bacterial ASVs from 1584 to 2053, demonstrating higher microbial richness. Principal Coordinates Analysis (PCoA) showed a clear separation between treatments, with PC1 explaining 76.1% of the variation, confirming that root enhancement markedly altered community structure. LEfSe analysis identified several discriminative bacterial taxa between treatments based on LDA scores, with Rhizobiales/Rhizobiaceae-affiliated taxa and Actinobacteria-related lineages showing higher contributions under the Nb treatment. Functional predictions indicated a higher predicted enrichment of nutrient-related functions—including ABC transporters (K02035), phosphate transport system proteins (K02032/K02034), and glutamine synthetase (K01952)—suggesting enhanced functional potential for microbial nutrient acquisition under the Nb treatment. Collectively, these results indicate that enhanced root development reshapes both enzyme activities and microbial functional potential, creating a more favorable biochemical and biological environment for plant growth.

### 3.5 Effects of root enhancement on agronomic traits and biomass accumulation

**Figure 7:**
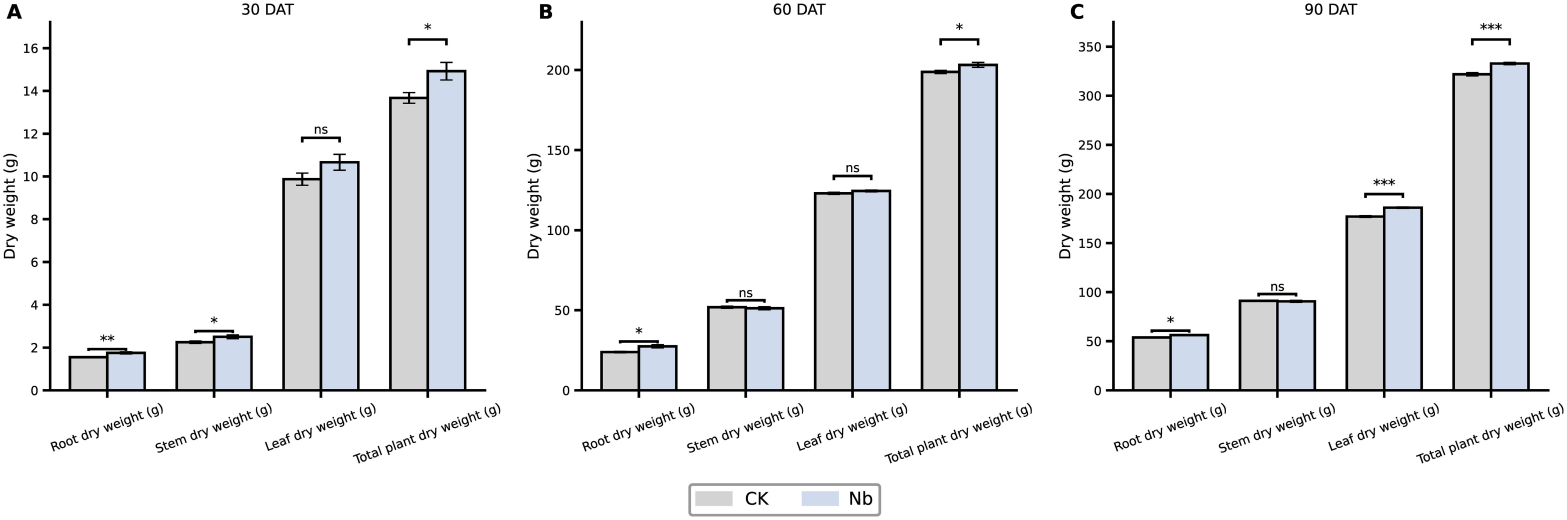
Effects of root enhancement on dry matter accumulation. Changes in root, stem, leaf, and total plant dry weight under different treatments at **(A)** 30, **(B)** 60, and **(C)** 90 days after transplanting. CK represents the control treatment, and Nb represents the treatment group. Error bars indicate standard error (SE). Statistical significance is denoted as *P < 0.05, **P < 0.01, ***P < 0.001, and ns indicates no significant difference.

**Figure 8:**
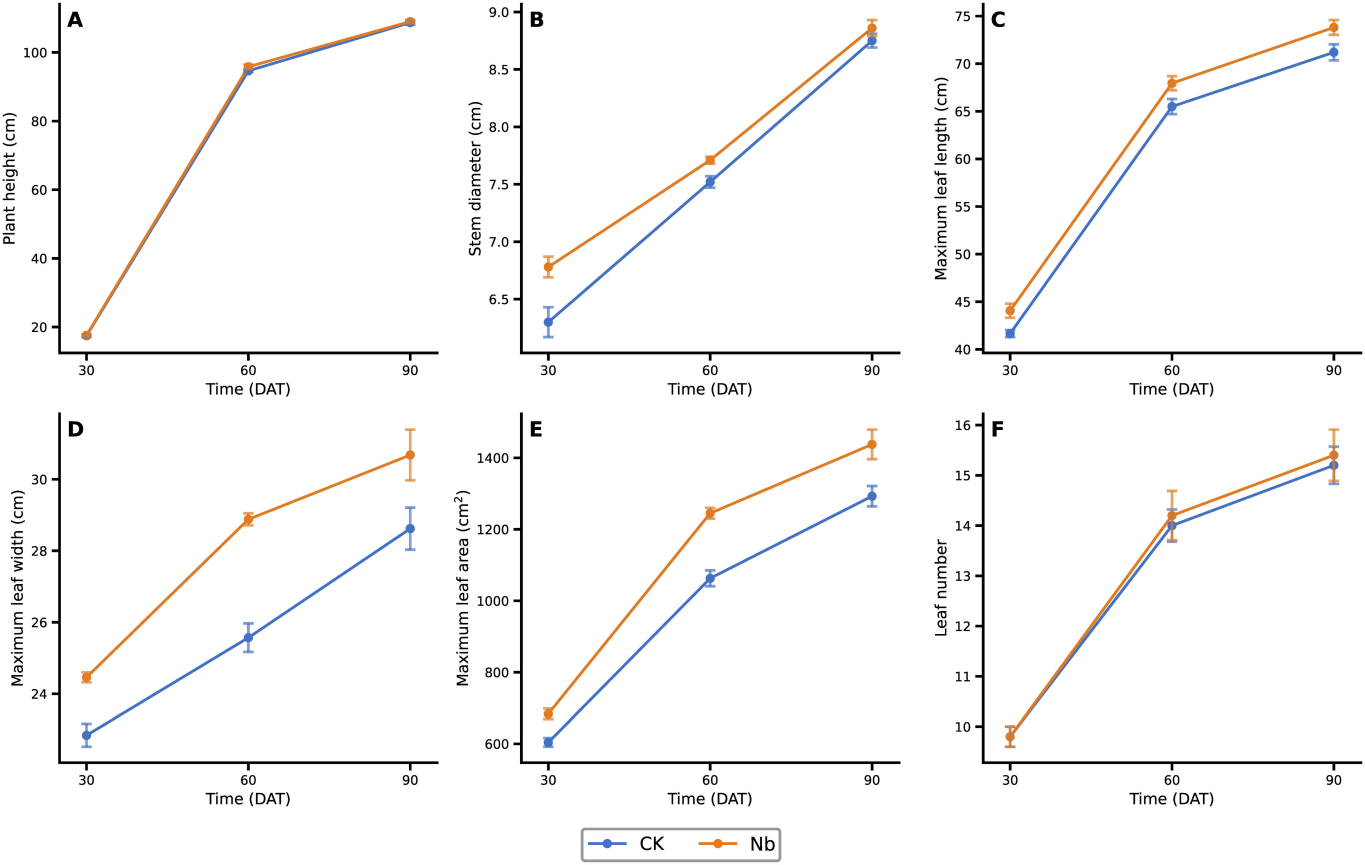
Effects of root enhancement on agronomic traits. Changes in **(A)** plant height, **(B)** stem diameter, **(C)** maximum leaf length, **(D)** maximum leaf width, **(E)** maximum leaf area, and **(F)** leaf number under different treatments at 30, 60, and 90 days after transplanting. CK represents the control treatment, and Nb represents the treatment group. Error bars indicate standard error (SE).

Leaf morphological development improved steadily under the Nb treatment during the field growth period (**Figure 7**). Maximum leaf area increased by 13.2%, 17.1%, and 11.2% at 30, 60, and 90 DAT, respectively, while maximum leaf width consistently exhibited a 7.1–12.9% advantage. Plant height showed no significant difference between treatments at any stage, indicating that enhanced root growth did not lead to excessive vegetative elongation. Biomass production also benefited from root enhancement (**Figure 8**). Total plant dry weight increased by 9.1%, 2.9%, and 2.8% at 30, 60, and 90 DAT, respectively. Among different organs, root dry weight displayed the most pronounced improvement, and leaf dry weight began to show a clear advantage by 90 DAT. Overall, the Nb treatment promoted a more favorable biomass allocation pattern—supporting greater leaf development without inducing unnecessary stem growth—thereby strengthening the plant’s capacity for sustained photosynthetic assimilation and dry matter accumulation.

### 3.6 Correlation analysis among variables

**Figure 9:**
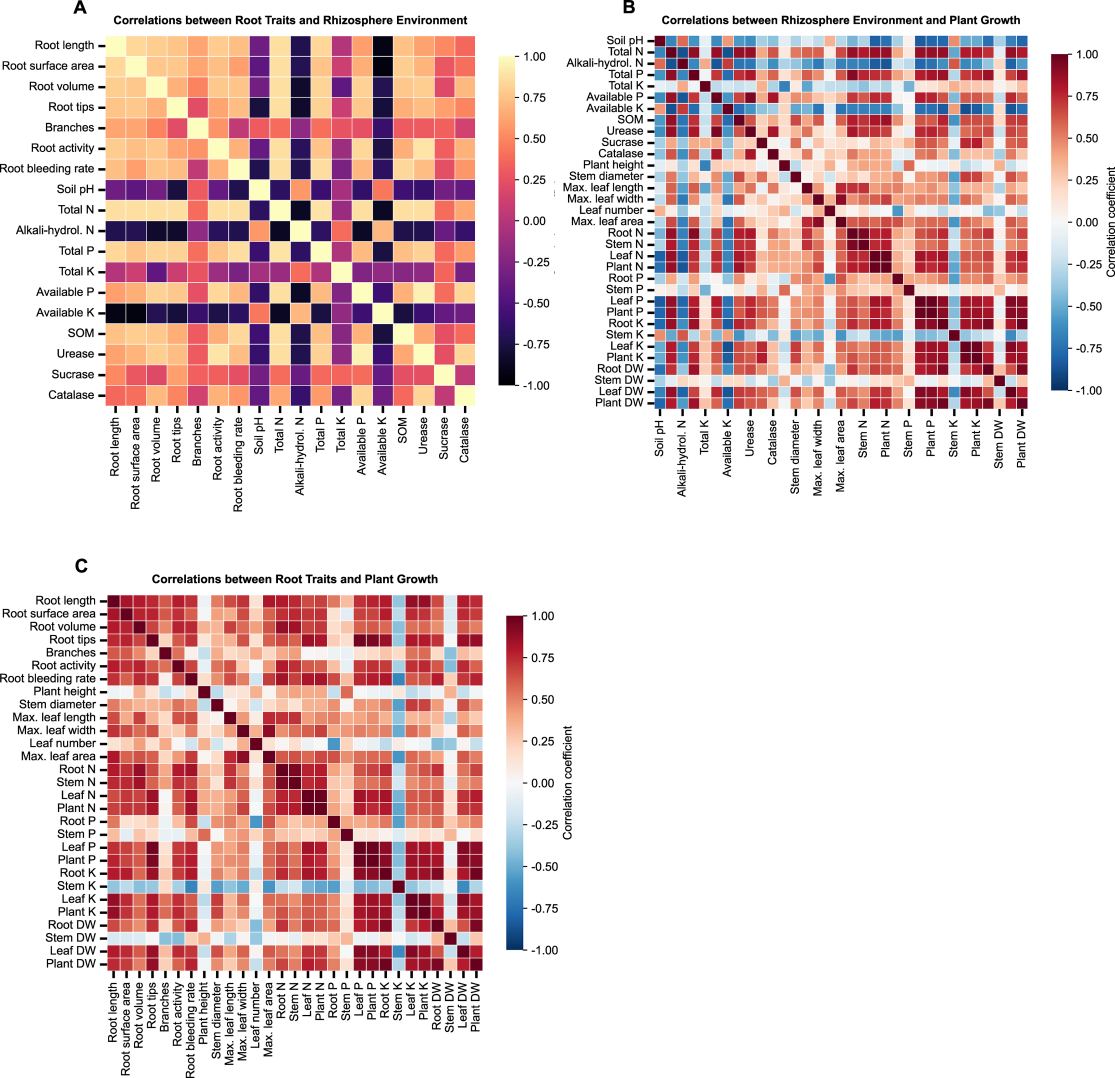
Correlation analysis among root traits, rhizosphere environment, and plant growth indicators. **(A)** Correlations between root traits and rhizosphere environmental factors; **(B)** correlations between rhizosphere environmental factors and plant growth indicators; **(C)** correlations between root traits and plant growth indicators. Pearson correlation coefficients are shown and represented by the color scale.

The correlation network revealed strong linkages between root traits, rhizosphere properties, and plant growth performance (**Figure 9**). Root length, surface area, volume, root activity, and exudation rate were all positively correlated with soil total nitrogen, total phosphorus, organic matter, urease activity, and available phosphorus. These relationships indicate that a more developed and physiologically active root system was associated with improved nutrient status and biochemical functioning of the rhizosphere. In contrast, these root traits showed significant negative correlations with soil available potassium and hydrolyzable nitrogen, suggesting that rapid nutrient uptake by the enhanced root system reduced the concentrations of these readily available nutrient pools in the rhizosphere. Plant performance closely followed the improvements in rhizosphere conditions. Soil total nitrogen, total phosphorus, organic matter, urease activity, and available phosphorus all showed strong positive correlations with total plant nutrient accumulation, leaf area expansion, and biomass production. Conversely, soil pH, hydrolyzable nitrogen, and available potassium were negatively correlated with these growth indicators, reflecting that lower pH and higher nutrient consumption were linked to improved plant development. Together, these correlation patterns highlight an integrated response in which enhanced root architecture and activity shape the rhizosphere environment, which in turn supports greater nutrient acquisition and plant growth.

### 3.7 Path analysis of the root–rhizosphere–plant system

**Figure 10:**
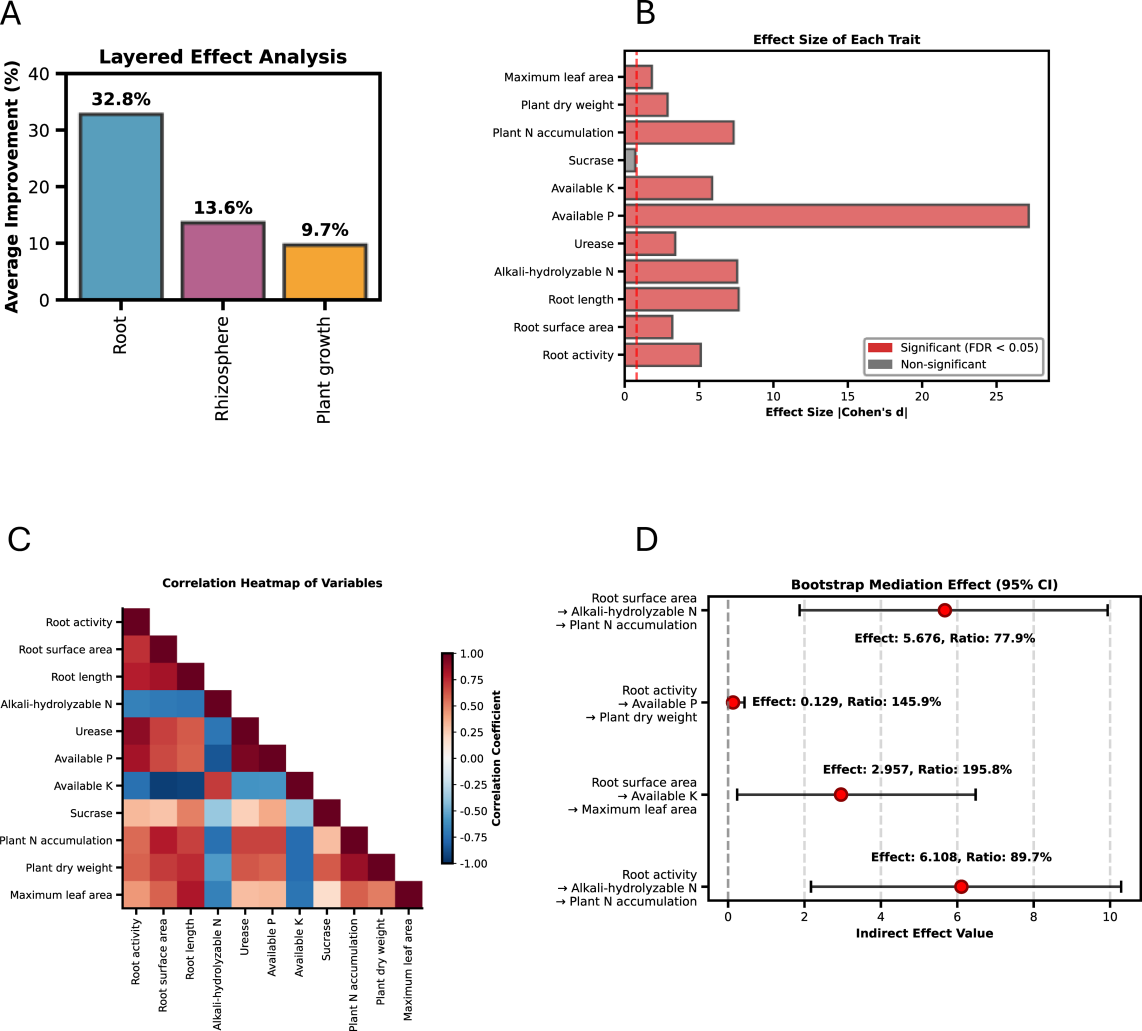
Integrated analysis of effect sizes, correlations, and mediation effects among root traits, soil environment, and plant growth indicators. **(A)** Layered effect analysis; **(B)** effect sizes of different traits (Cohen’s d); **(C)** correlation heatmap of variables; **(D)** bootstrap-based mediation analysis with 95% confidence intervals.

**Figure 11:**
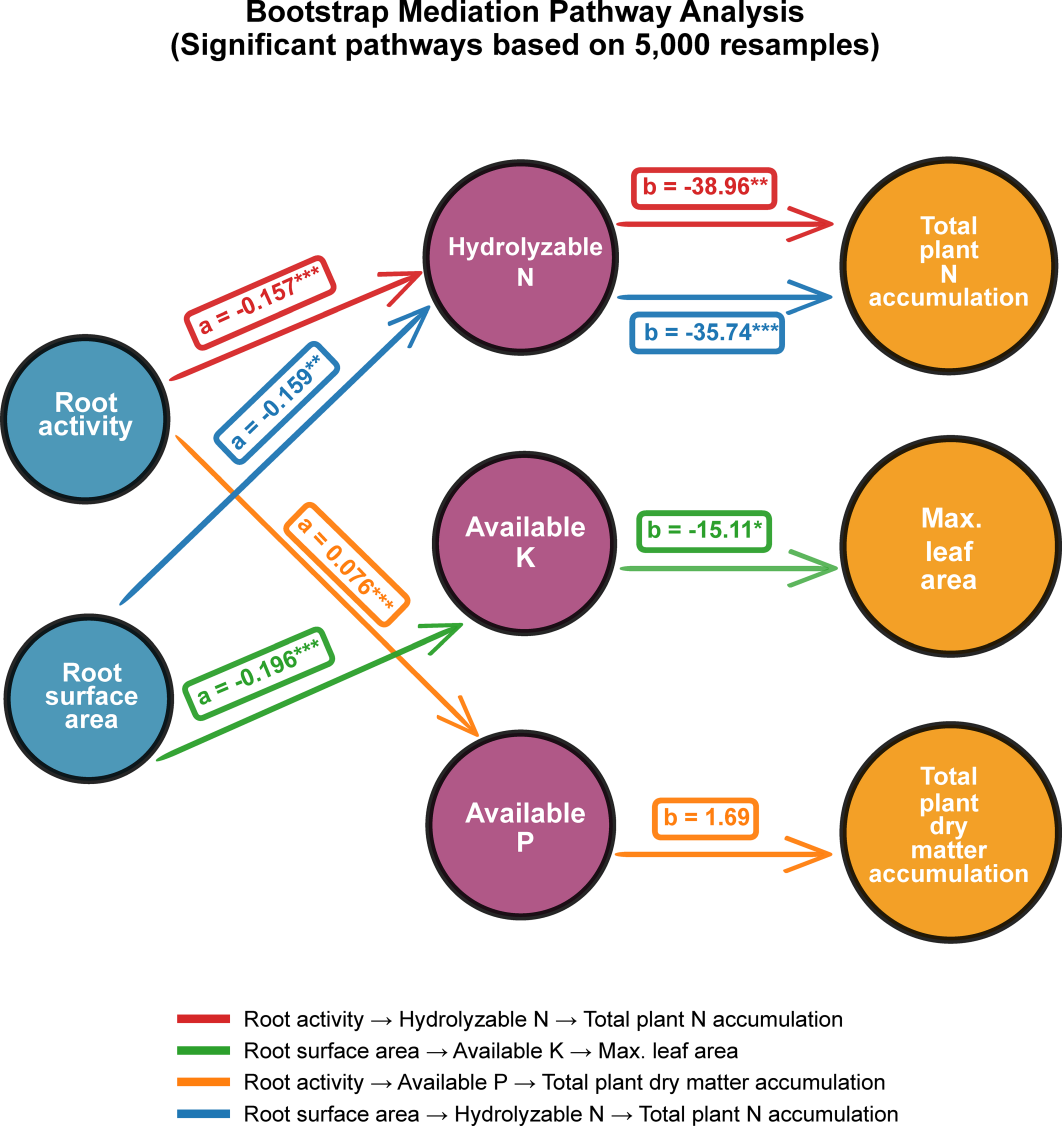
Bootstrap mediation pathway diagram. Bootstrap mediation analysis based on 5,000 resamples, showing only significant mediation pathways (P < 0.05). Path coefficient a represents the effect of the independent variable (X) on the mediator, while b represents the effect of the mediator on the dependent variable (Y); the indirect effect is calculated as a × b. All pathways represent full mediation effects. Statistical significance is denoted as *P < 0.05, **P < 0.01, and ***P < 0.001.

To clarify how root enhancement influences plant performance through rhizosphere processes, we integrated differential analysis with a mediation-effect framework (**Figure 10**). Among the 11 measured variables, 10 exhibited significant treatment responses after FDR correction, and 7 remained significant under the stricter Bonferroni adjustment, confirming that the Nb treatment generated coordinated shifts across root, soil, and plant layers. Mediation analysis identified four significant pathways, all showing full mediation (**Figure 11**). Two pathways linked root traits to plant nitrogen accumulation through rhizosphere nitrogen availability. The total effect of root activity on total plant N accumulation (c = 6.812) was almost entirely transmitted through soil hydrolyzable nitrogen (indirect effect = 6.108; 95% CI: 2.170–10.289), accounting for 89.7% of the effect. A similar full mediation was observed for root surface area, where soil hydrolyzable nitrogen explained 77.9% of its total effect on plant N accumulation (indirect effect = 5.676; 95% CI: 1.872–9.936). Two additional pathways exhibited suppression effects, in which indirect and direct effects acted in opposite directions. Root activity increased total plant dry weight primarily through its positive effect on soil available phosphorus (indirect effect = 0.129; 95% CI: 0.003–0.429). Likewise, root surface area enhanced maximum leaf area via reduced soil available potassium (indirect effect = 2.957; 95% CI: 0.237–6.481). In both cases, the indirect effect outweighed the opposing direct effect, resulting in overall significant positive impacts. Collectively, mediation analysis identified four statistically significant pathways linking root traits with plant performance, all of which showed full mediation by rhizosphere variables. The magnitudes and directions of indirect effects differed among nitrogen, phosphorus, and potassium-related pathways, and suppression effects were observed in the phosphorus–biomass and potassium–leaf area pathways. These results indicate that the effects of root traits on plant growth metrics were predominately transmitted through measured rhizosphere factors.

## 4 Discussion

### 4.1 Effects of root enhancement on the rhizosphere environment

#### 4.1.1 Effect of root enhancement on soil nutrient activation

The Nb treatment substantially strengthened the root system architecture, particularly by increasing the density of root tips and branches, which expanded both the physical contact area and the biochemical influence of roots on the surrounding soil (Griffiths et al., 2022; Lynch, 2022). A more complex and metabolically active root system generally releases greater quantities of organic acids and protons, thereby enhancing rhizosphere acidification. This trend aligned with the significant pH decline observed at 60 DAT, a response consistent with root-driven acidification mechanisms reported in previous studies (Galatro et al., 2020; Fujii et al., 2021). Such acidification promotes the dissolution of insoluble phosphate minerals, explaining the sharp increase in available phosphorus under Nb treatment (Gámez-Arjona et al., 2022; Santoro et al., 2024). It should be noted that the pronounced relative increase in available phosphorus cannot be attributed to rhizosphere acidification alone (Staunton and Pistocchi, 2025). The Nb treatment significantly enriched phosphorus-mobilizing microorganisms, including Rhizobiaceae and Actinobacteria, which are known to accelerate phosphate solubilization and mineral dissolution through organic acids and enzymatic pathways (Walpola and Yoon, 2012; Gupta et al., 2024). Functional predictions further showed upregulation of phosphate-transport proteins (K02032/K02034) and ABC transport systems, indicating enhanced microbial capacity for P acquisition and turnover (Li et al., 2024; Zhou et al., 2025). In addition, the baseline AP concentration of the soil was relatively low, meaning that a modest absolute increase translated into a large percentage change, a pattern commonly observed in low-P environments (Liu et al., 2019). Therefore, the observed AP increase represents the synergistic action of root-induced acidification, microbial P mobilization, and low initial soil P status, rather than a sole pH effect. The Nb treatment also affected nitrogen dynamics in a time-dependent manner. Hydrolyzable nitrogen increased during the early and mid-growth periods but declined by 90 DAT, indicating that plant uptake eventually exceeded the mineralization rate. This pattern is consistent with evidence that vigorously growing roots secrete amino acids and organic acids capable of stimulating urease activity and accelerating nitrogen transformation (Sardans et al., 2023). Available potassium exhibited a similar temporal pattern: a slight early increase, stabilization at mid-stage, and depletion by 90 DAT. Organic acids released by roots can displace K^+^ from soil colloids and enhance the weathering of K-bearing minerals (Yang et al., 2019). However, when root uptake surpasses the soil’s replenishment capacity, local rhizosphere K depletion can occur, as reflected at 90 DAT and supported by prior observations (Volf et al., 2018). In addition, the Nb treatment caused a transient increase in soil organic matter at the early sampling stage. The absolute difference was relatively small but appeared large when expressed as a percentage because the background SOM level was moderate. Given that our sampling focused on the immediate rhizosphere, this early enrichment most likely reflects enhanced rhizodeposition—including root exudates, sloughed root tissues, and microbial necromass—rather than a fundamental shift in bulk soil organic matter (Jones et al., 2004, 2009). This interpretation is supported by the progressive decline in the Nb–CK difference to 8.8% at 60 DAT and 4.6% at 90 DAT, indicating that part of the labile carbon pool was rapidly turned over by microorganisms. Because dichromate oxidation is particularly sensitive to recently added, biologically active organic carbon fractions (Sun et al., 2016), the observed difference mainly reflects short-term rhizosphere “hotspots” rather than long-term changes in background SOM. Collectively, these patterns indicate that improvements in root architecture under the Nb treatment acted as a primary driver of rhizosphere nutrient activation, with distinct early- and late-stage dynamics shaping N, P, K availability and short-term organic matter hotspots.

#### 4.1.2 Effect of root enhancement on soil enzyme activities

Soil enzyme activities reflect the biochemical intensity of nutrient cycling in the rhizosphere and are tightly regulated by root exudation and microbial metabolism. In this study, the Nb treatment substantially promoted root morphogenesis and root physiological activity, thereby supplying more substrates and inducers for soil enzymatic reactions (Adamczyk et al., 2021; Kern-Cardoso et al., 2022). Urease activity, a key indicator of nitrogen transformation, increased significantly at both early and late growth stages, showing strong positive correlation with root activity. This agrees with the central role of urease in facilitating N mineralization pathways and its sensitivity to changes in rhizosphere biochemical inputs (Zhang et al., 2023). Sucrase activity peaked at 60 DAT, which coincided with the period of rapid root growth and intensified rhizodeposition. As well-developed root systems typically release higher quantities of soluble sugars and carbon compounds, they provide substrates that stimulate microbial proliferation and carbon-cycling enzyme systems (Zhalnina et al., 2018; Ma et al., 2022b). Catalase activity exhibited a moderate but significant increase, reflecting adjustments to the oxidative environment of the rhizosphere. Catalase decomposes reactive oxygen species (ROS) generated during rapid root metabolism or elevated microbial turnover, and its activation helps maintain redox balance and biochemical stability (Bissaro et al., 2018; Chang et al., 2018). The increase in root metabolic intensity and secretion of antioxidant compounds under Nb treatment may have induced this response, which aligns with previous observations that the Nb treatment can influence rhizosphere ROS dynamics (Canarini et al., 2019). Collectively, these results demonstrate that root architectural and functional optimization amplifies the biochemical activity of the rhizosphere by stimulating key C- and N-cycling enzyme systems, strengthening the micro-ecological foundation for nutrient transformation.

#### 4.1.3 Effect of root enhancement on the structure and functional potential of the rhizosphere microbial community

A structurally enhanced root system substantially reshaped the rhizosphere microbial community, as evidenced by the clear divergence in community composition between treatments. Principal Coordinate Analysis revealed that PC1 alone explained 76.1% of the variation, demonstrating that the microbial shift induced by root optimization was not a stochastic alteration but rather a highly selective recruitment process shaped by root-derived chemical signals and substrate inputs (Zhalnina et al., 2018; Balasubramanian et al., 2020). This finding aligns with the understanding that root systems with greater surface area and metabolic activity exert stronger biochemical filtering effects on their surrounding microbiota. The functional prediction analysis further clarified how the Nb treatment influenced microbial ecological roles. The Nb treatment significantly upregulated several key pathways—including the ABC transport system (K02035), phosphate transport proteins (K02032/K02034), and glutamine synthetase (K01952)—all of which are central to microbial nutrient acquisition, energy transfer, and environmental adaptability. ABC transporters play vital roles in the active translocation of organic acids, amino acids, and other small molecules, enabling microorganisms to maintain metabolic homeostasis under fluctuating rhizosphere conditions (Young et al., 2006; Rismondo and Schulz, 2021). Similarly, the elevated abundance of phosphate transport proteins suggests that microorganisms in the Nb rhizosphere possessed a stronger capacity for inorganic phosphorus acquisition, consistent with the observed increase in soil available P and supporting the notion of a cooperative plant–microbe phosphorus activation network (Solangi et al., 2023). The enhanced expression of glutamine synthetase further implies intensified microbial participation in nitrogen cycling. Microorganisms with high glutamine synthetase activity exhibit strong ammonium assimilation capacity, contributing to improved nitrogen use efficiency within plant–microbe symbioses (Sandhu et al., 2021; Ghosh et al., 2024). Taken together, these functional shifts indicate that root architecture optimization not only reshaped microbial composition but also steered the community toward nutrient-mobilizing and nutrient-efficient functional groups, thereby providing biological support for improved plant nutrient uptake.

It is important to note, however, that the present functional predictions were derived from marker-gene data. Although these results provide meaningful ecological insights, the underlying mechanisms of microbial metabolic restructuring cannot be fully resolved without complementary multi-omics analyses, such as metagenomics, metabolomics, and transcriptomics. Future studies integrating these approaches will be essential for confirming the biochemical pathways through which root enhancement regulates rhizosphere microbial functions.

### 4.2 Effects of the rhizosphere environment on plant growth

#### 4.2.1 Effect of the rhizosphere environment on plant nutrient uptake

A well-functioning rhizosphere environment serves as the essential intermediary linking root optimization to improved nutrient acquisition and plant growth (Wang et al., 2023; Grondin et al., 2024).In this study, the Nb treatment markedly enhanced key rhizosphere properties, including available phosphorus and urease activity, both of which remained consistently higher than the control through 90 DAT. Correspondingly, total plant nitrogen and phosphorus accumulation increased by 13–14%, indicating that improvements in the rhizosphere were effectively translated into enhanced nutrient uptake. These findings are consistent with previous research showing that root-driven rhizosphere modification—particularly through exudation and biochemical activation—plays a central role in facilitating nutrient mobilization and uptake (Oburger et al., 2022; Sun et al., 2024). An intriguing pattern emerged in the negative correlations between root development indicators (root length, root surface area) and the concentrations of available potassium and hydrolyzable nitrogen. Although seemingly paradoxical, similar phenomena have been reported previously (Jing et al., 2021; Lopez et al., 2023). This pattern reflects a physiological process rather than a measurement artifact. A more extensive and physiologically active root system can absorb readily available nutrients at a rate that exceeds the soil’s natural replenishment capacity, thereby temporarily reducing their concentrations in the rhizosphere (Ren et al., 2023). In other words, diminished soil nutrient concentrations are a signature of accelerated uptake, not nutrient scarcity. This interpretation is further supported by the nutrient accumulation results. For example, the strong negative correlation between root surface area and soil available potassium contrasts with the overall increase in plant potassium accumulation. This suggests that the enlarged root absorption area enhanced potassium acquisition efficiency, even though the rhizosphere potassium concentration decreased (Hinsinger et al., 2021). Together, these findings demonstrate that improvements in root architecture reshape the rhizosphere in a manner that strengthens nutrient acquisition, supporting the conclusion that the rhizosphere environment is a central mediator of plant nutrient uptake.

#### 4.2.2 Effect of the rhizosphere environment on above-ground plant parts

Interestingly, although the Nb treatment did not significantly increase plant height, it substantially promoted leaf development. At 90 DAT, the maximum leaf area under the Nb treatment was 11.2% higher than that of the control, indicating a distinct shift in above-ground growth patterns. This type of growth adjustment merits further examination because an adequate nutrient supply often leads to excessive vegetative elongation in many crops (Parisi et al., 2022). However, in the present study, plants under improved rhizosphere conditions appeared to adopt a more “economical” allocation strategy—investing preferentially in the expansion of photosynthetic organs rather than increasing stem height. Such a shift is consistent with ecological theory. It has been reported that when soil resource acquisition becomes more efficient, plants often transition from a “conservative” strategy characterized by long leaf lifespan and slow carbon turnover to a more “acquisitive” strategy marked by enhanced photosynthetic efficiency and rapid resource utilization (Klimešová et al., 2021). This transition typically results from improved nutrient uptake efficiency and reduced below-ground competitive pressure. Moreover, resource allocation is also shaped by above-ground competition. Under favorable rhizosphere conditions, the need for height-mediated light competition is reduced, and plants tend to allocate more biomass toward leaf expansion and chlorophyll enrichment to maximize photosynthetic returns (Wang et al., 2020; Dong et al., 2023). Similar findings have been reported in studies emphasizing that improved below-ground nutrition allows plants to optimize photosynthetic structures rather than expend energy on unnecessary stem elongation (Lammerts van Bueren and Struik, 2017; Wang et al., 2022b). This optimization of growth strategy ultimately led to a steady increase in biomass. At 90 DAT, the total plant dry weight and leaf dry weight increased by 2.8% and 4.1%, respectively. Although the magnitude of the increase appears modest, these values represent the cumulative outcome of resource reallocation over the full growth cycle. Such consistent and stable improvement in above-ground productivity is likely to exert positive effects on later developmental stages, ultimately contributing to enhanced field performance.

### 4.3 Verification and interpretation of the root-soil-plant regulatory mechanism

#### 4.3.1 Identification of major regulatory pathways

Mediation analysis revealed four statistically significant regulatory pathways within the “root–rhizosphere–plant” system. Several other potential pathways—such as those involving sucrase or root length as mediators—did not meet the statistical threshold and were therefore excluded from the discussion of primary mechanisms. It is worth noting that although some pathways were not significant after the stringent Bonferroni correction, they remained significant under the False Discovery Rate (FDR) correction. Given that the FDR approach offers greater statistical power while still effectively controlling Type I error, the present study adopted FDR-corrected results to identify the major regulatory pathways. Nitrogen regulatory pathway: Two pathways—”Root activity → Hydrolyzable N → Total plant N accumulation” and “Root surface area → Hydrolyzable N → Total plant N accumulation”—showed strong indirect effects, accounting for 89.7% and 77.9% of the total effect, respectively. These results indicate that the influence of the Nb treatment—via improved root architecture—on plant nitrogen nutrition is primarily transmitted via its modulation of the rhizosphere nitrogen supply rather than through a direct pathway, consistent with previous findings on rhizosphere-mediated nitrogen acquisition (Zhu et al., 2021). Phosphorus and biomass regulatory pathway: For the pathway “Root activity → Available P → Total plant dry weight,” the mediated (indirect) effect accounted for 145.9% of the total effect. This is characteristic of a suppression effect, wherein direct and indirect effects act in opposite directions, causing the proportion of the mediated effect to exceed 100% (MacKinnon et al., 2007; Bolin, 2014). This indicates that the biomass-promoting effect of root activity is predominantly dependent on its ability to enhance rhizosphere phosphorus availability. Root activity reflects metabolic intensity, and highly active roots typically release greater quantities of organic acids and other exudates that promote the activation of poorly soluble phosphorus fractions (Ma et al., 2022a; Pang et al., 2024). Considering the indispensable role of phosphorus in energy metabolism, cellular signaling, and biosynthesis (Bossa et al., 2024), rhizosphere P activation emerges as a crucial mechanism supporting improved plant growth. Potassium and leaf development pathway: Similarly, the pathway “Root surface area → Available K → Maximum leaf area” exhibited a mediated effect proportion of 195.8%, another example of a suppression effect. The direct effect was negative, whereas the indirect effect was strongly positive and dominated the overall influence. This reflects the element-specific roles of root traits: root activity primarily participated in nitrogen and phosphorus cycling, whereas root surface area exerted stronger control over potassium dynamics. Because potassium is predominantly transported in soil via diffusion, increases in root surface area—which enhance soil–root contact—can disproportionately improve potassium uptake efficiency (Huai et al., 2022). This efficiently supports the expansion of leaf area, a key determinant of photosynthetic capacity. Overall, the identified pathways highlight distinct nutrient-specific mediation routes. These results quantitatively confirm that the impact of root enhancement on plant performance is transmitted through targeted modifications of nitrogen, phosphorus, and potassium availability in the rhizosphere.

#### 4.3.2 Interpretation of the regulatory mechanism

The mediation analysis demonstrated that the rhizosphere environment is the pivotal intermediary linking the Nb treatment to plant nutrient uptake and biomass accumulation. This finding reinforces the concept that evaluating root performance based solely on morphological traits is insufficient; rather, attention must be directed toward the quality and functionality of the rhizosphere environment, which ultimately determines the effectiveness of nutrient acquisition (Xue et al., 2016). The nutrient-specific pathways revealed distinct regulatory characteristics. Notably, both the phosphorus and potassium pathways exhibited indirect effect proportions exceeding 100%, indicative of suppression effects. This pattern suggests that although the Nb treatment may exert a weak competitive or negative direct effect on above-ground organs, the positive indirect effect mediated through rhizosphere nutrient activation far outweighs the direct effect, resulting in an overall promotion of plant growth. This provides a novel perspective for understanding how plants balance resource allocation between below-ground and above-ground structures while maintaining efficient nutrient use. From a cultivation perspective, the verified root–rhizosphere–plant mechanism offers a solid theoretical foundation for management strategies (Zhou et al., 2023b). The results clearly indicate that to promote plant performance, agronomic interventions should prioritize methods that improve the rhizosphere environment, rather than focusing exclusively on stimulating root growth (Shen et al., 2013; Sarkar, 2021). Enhancing the rhizosphere’s nutrient cycling capacity, microorganism-supported nutrient conversion, and biochemical activation appears to be central to sustaining long-term increases in nutrient uptake and biomass formation. Although mediation analysis revealed statistically supported causal relationships, the underlying biological processes remain to be fully elucidated. Key questions persist, such as how specific root traits regulate nutrient activation pathways within the rhizosphere, and whether different nutrients exhibit unique spatial or temporal activation patterns. Addressing these questions through future mechanistic studies will deepen our understanding of root functional biology and its role in shaping plant–soil interactions.

## 5 Conclusion

In conclusion, the optimization of root architecture and function in flue-cured tobacco is a key driving force for regulating the rhizosphere environment and promoting plant growth and development. Structurally and functionally enhanced root systems effectively improved the rhizosphere microenvironment by lowering soil pH, increasing key enzyme activities, and reshaping the microbial community structure, which significantly enhanced the availability of nutrients such as available phosphorus. The improved rhizosphere environment, in turn, promoted the uptake and accumulation of nitrogen, phosphorus, and potassium by the plant, led to a preferential allocation of resources to the leaves, and ultimately increased the total plant biomass. Mediation analysis confirmed that the rhizosphere environment plays a crucial, fully mediating role in the causal chain from “root optimization” to “plant growth.” These improvements were achieved through the nutrient-bag (Nb) seedling cultivation technique, which consistently promoted root architectural and physiological optimization during field growth. Therefore, optimizing the root system is an effective pathway to enhance the field performance of flue-cured tobacco by regulating the rhizosphere environment.

## Data Availability

The datasets presented in this study can be found in online repositories. The names of the repository/repositories and accession number(s) can be found in the article/**Supplementary Material**.
