# Supplementary material for: Root enhancement improves rhizosphere nutrient availability and promotes growth in flue-cured tobacco
Source: Front Plant Sci. 2026 Jan 15;16:1728181. doi: 10.3389/fpls.2025.1728181 (PMC12854075; doi:10.3389/fpls.2025.1728181)
Supplement: Supplementary file 1 [file DataSheet1.docx]

Supplementary Material

# Supplementary Figures and Tables

## Supplementary Figures


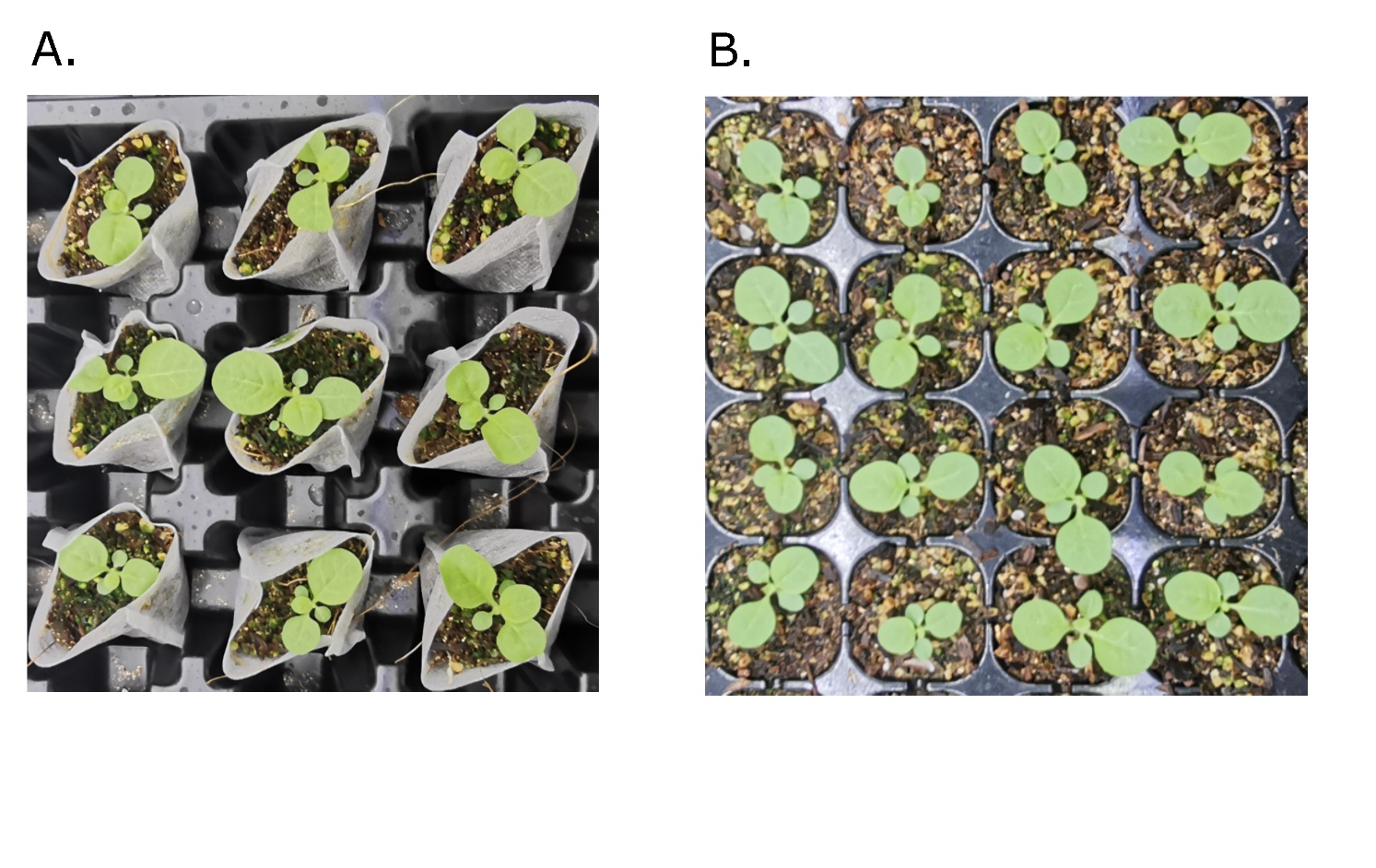


**Supplementary Figure 1. Photos of seedlings**

Conventional Floating Seedling Cultivation (CK, Fig.B) and Root-Enhancement Seedling Cultivation (Nb, Fig.A).


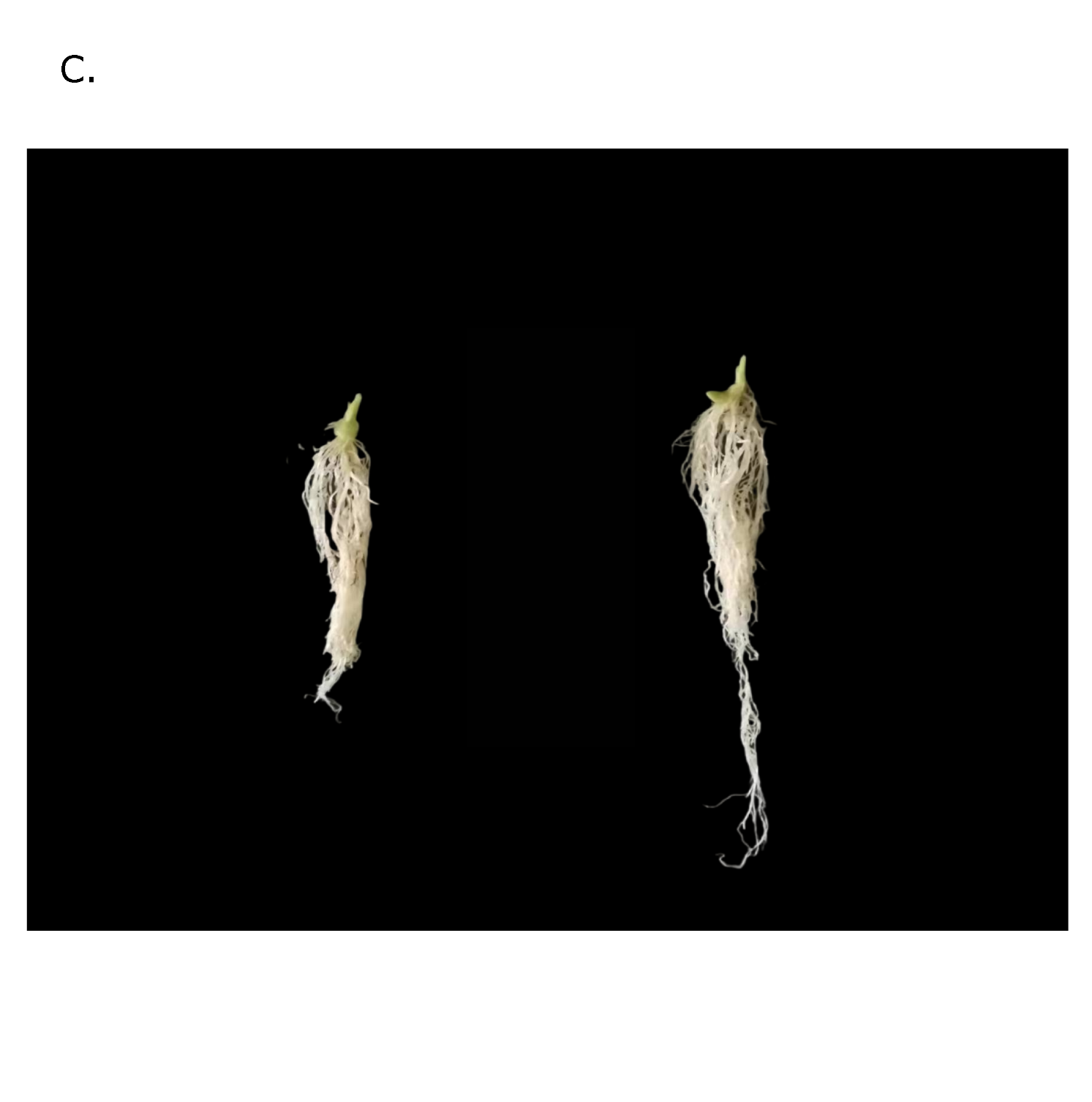


**Supplementary Figure 2. Photos of root systems**

Root system morphology of Conventional Floating Seedling Cultivation (CK, left) and Root-Enhancement Seedling Cultivation (Nb, right).


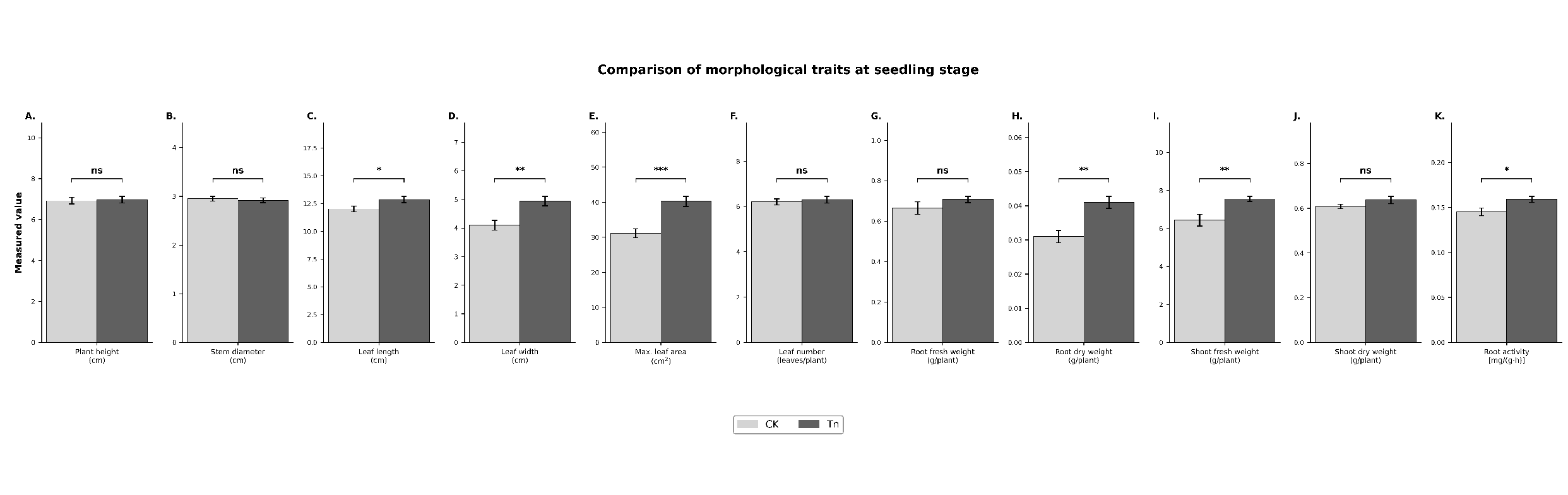


**Supplementary Figure 3. Comparison of morphological traits at the seedling stage.**

Comparisons of (A) plant height, (B) stem diameter, (C) leaf length, (D) leaf width, (E) maximum leaf area, (F) leaf number, (G) root length, (H) root dry weight, (I) stem dry weight, (J) leaf dry weight, and (K) root activity under different treatments. CK represents the control treatment, and Tn represents the treatment group. Error bars indicate standard error (SE). Statistical significance is denoted as * p < 0.05, ** p < 0.01, *** p < 0.001, and ns indicates no significant difference.

## Supplementary Tables

**Supplementary Table 1. Agronomic traits, biomass, and root activity of tobacco seedlings during the rooting stage**

| Parameter | CK | Nb | P-value | Significance |
| --- | --- | --- | --- | --- |
| Plant height (cm) | 6.92 ± 0.51 | 6.98 ± 0.53 | 0.815 | ns |
| Stem girth (cm) | 2.95 ± 0.16 | 2.92 ± 0.15 | 0.618 | ns |
| Leaf length (cm) | 12.02 ± 0.84 | 12.86 ± 0.94 | 0.049 | * |
| Leaf width (cm) | 4.09 ± 0.53 | 4.94 ± 0.54 | 0.002 | ** |
| Maximum leaf area (cm²) | 31.17 ± 4.01 | 40.25 ± 4.62 | < 0.001 | *** |
| Number of leaves (leaves/plant) | 6.20 ± 0.42 | 6.30 ± 0.48 | 0.651 | ns |
| Below-ground fresh weight (g/plant) | 0.66 ± 0.10 | 0.71 ± 0.05 | 0.230 | ns |
| Below-ground dry weight (g/plant) | 0.03 ± 0.01 | 0.04 ± 0.01 | 0.002 | ** |
| Above-ground fresh weight (g/plant) | 6.43 ± 0.97 | 7.55 ± 0.44 | 0.004 | ** |
| Above-ground dry weight (g/plant) | 0.61 ± 0.03 | 0.64 ± 0.05 | 0.155 | ns |
| Root activity [mg/(g·h)] | 0.15 ± 0.01 | 0.16 ± 0.01 | 0.025 | * |

CK represents the control treatment, and Nb represents the treatment group. Significance levels: ns, not significant; *, *P* < 0.05; **, *P* < 0.01; ***, *P* < 0.001.
